# Predictors of Physical Activity Enjoyment in Adults with Cystic Fibrosis: The Role of Quality of Life and Motivation—A Single-Center Study

**DOI:** 10.3390/healthcare13172194

**Published:** 2025-09-02

**Authors:** Wolfgang Gruber, Florian Stehling, Jin-Sun Schermaul, Jose G. Ortiz, Liron Lechtenberg, Christian Taube, Matthias Welsner

**Affiliations:** 1 Pediatric Pulmonology and Sleep Medicine, Cystic Fibrosis Center, Children’s Hospital, University of Duisburg-Essen, 45147 Essen, Germany; 2 Department of Pulmonary Medicine, University Hospital Essen—Ruhrlandklinik, Adult Cystic Fibrosis Center, University of Duisburg-Essen, 45147 Essen, Germany

**Keywords:** cystic fibrosis, physical activity, enjoyment, motivation, health-related quality of life

## Abstract

**Background:** Despite the well-documented physical and psychological benefits of regular physical activity (PA) and exercise, participation remains insufficient in adults with cystic fibrosis (pwCF). In the general population, PA enjoyment is a key determinant of sustained engagement, yet its predictors in CF populations remain underexplored. **Objective:** We aimed to examine associations between clinical parameters, health-related quality of life (HRQoL), motivation and PA enjoyment in adult pwCF. We hypothesised that higher intrinsic motivation and better HRQoL would predict greater enjoyment, independent of clinical parameters. **Methods:** In this cross-sectional study, 197 adult pwCF (mean age = 36.6 ± 11.9 years) from a single centre completed validated questionnaires assessing PA and exercise enjoyment (Physical Activity Enjoyment Scale, PACES), motivation (Behavioral Regulation in Exercise Questionnaire-2, BREQ-2), and HRQoL (Cystic Fibrosis Questionnaire-Revised, CFQ-R). Hierarchical regression was conducted in three steps: clinical variables (Model 1), added HRQoL domains (Model 2), and motivational variables (Model 3). **Results**: The complete model explained 68.4% of the variance in PA and exercise enjoyment (R^2^ = 0.684, *p* < 0.001). Intrinsic motivation was the strongest positive predictor (β = 6.228, *p* < 0.001), while external regulation negatively predicted enjoyment (β = −1.932, *p* = 0.030). Among HRQoL domains, only health perception remained significant (β = 0.081, *p* = 0.038). Clinical variables alone accounted for minimal variance (R^2^ = 0.023, *p* = 0.370). **Conclusions:** Intrinsic motivation was the most robust predictor of PA and exercise enjoyment, outweighing clinical and most HRQoL factors. These findings support autonomy-supportive strategies to foster internal motivation and enhance long-term PA and exercise participation in adult pwCF.

## 1. Introduction

Regular physical activity (PA) and exercise are fundamental elements of evidence-based care for people with cystic fibrosis (pwCF). Both aerobic and resistance exercises have been shown to improve cardiorespiratory fitness, muscle strength, and bone mineral density, all of which are commonly impaired in pwCF. Importantly, regular exercise has been shown to correlate with a slower decline in pulmonary function, better prognosis and improved health-related quality of life (HRQoL) [1,2,3].

Despite these well-documented benefits, engagement in PA among pwCF remains highly variable and frequently inadequate. Physical inactivity exacerbates pulmonary deterioration, reduces muscle mass and bone density, and compromises cardiovascular health in pwCF. Moreover, sedentary behavior accelerates deconditioning, increases breathlessness during daily activities and perpetuates the decline in PA [2].

Given the importance of PA and regular exercise for pwCF, it is crucial to understand barriers and facilitators to participation. Barriers to participation include the burden of treatment, fatigue, breathlessness, fear of pulmonary exacerbations, poor health due to CF, lack of time or appropriate facilities, and other physical, logistical and social factors. In contrast, perceived competence, social support, a favourable environment, and structured programs have been shown to improve adherence to regular and sustained PA and exercise in people with CF [4,5,6,7,8].

Beyond these physical and logistical considerations, psychological variables such as motivation and affective responses such as enjoyment, appear to play a significant role in shaping exercise behaviour [4,5,6]. Research in the general population has consistently demonstrated that enjoyment, along with other factors such as self-efficacy and motivation, exerts a substantial influence on exercise participation and maintenance [7,8,9].

Exercise and PA enjoyment can be defined as a positive affective response to movement reflecting feelings such as enthusiasm and excitement resulting from the activity itself [9,10]. Furthermore, individuals who are intrinsically motivated to engage in PA and exercise are more likely to experience higher levels of enjoyment, significantly influencing their engagement and commitment to PA and exercise [7,9,10].

Self-Determination Theory (SDT) provides a comprehensive framework for understanding these motivational processes and their impact on behaviour and well-being [8,11,12]. According to SDT, human motivation exists on a continuum that varies from amotivation through various forms of extrinsic motivation to intrinsic motivation. The theory posits that satisfaction of three basic psychological needs—autonomy (feeling volitional), competence (feeling effective), and relatedness (feeling connected)—promotes more autonomous forms of motivation, which are associated with greater persistence, engagement, and well-being [8]. In healthcare contexts, SDT has demonstrated that intrinsically motivated patients show better treatment adherence and outcomes than those who are motivated by external pressures or obligations [13,14].

While psychological factors are important determinants of exercise enjoyment, clinical characteristics such as disease severity (ppFEV_1_), demographics, and treatment status may also influence affective responses to physical activity and exercise. Understanding the relative contributions of clinical versus psychological predictors is essential for developing targeted interventions [2,5].

While these relationships are well-established in healthy populations, research on enjoyment and its relationship to PA and exercise participation in pwCF remains scarce. The limited available data suggest that emotional experiences, such as enjoyment, along with physical capability, significantly impact PA and exercise participation and maintenance in pwCF [1,4,5,6,15,16].

As health-related quality of life (HRQoL) is often reduced in pwCF due to the impact of the disease and the demands of treatment, it is important to understand how enjoyment influences exercise andPA [17,18,19]. Although Giannakoulakos et al. observed a positive correlation between PA and HRQoL, their study did not examine how exercise enjoyment and motivation influence various HRQoL domains, nor whether these domains reciprocally affect motivation and enjoyment [18].

The introduction of highly effective CFTR modulators, most notably the triple combination elexacaftor/tezacaftor/ivacaftor (ETI), has transformed CF management. Medical treatments have substantially improved lung function, nutritional status, health-related quality of life (HRQoL), and, in some cases, exercise capacity [20,21,22]. As these therapies may alter perceived health status and self-efficacy in people with CF (pwCF), it is increasingly important to assess their impact on exercise-related psychological outcomes in CF care. However, to date, no studies have examined their effects on PA motivation and enjoyment.

In this study, we aim to investigate the predictors of exercise enjoyment in adults with CF, examining the relative contributions of clinical factors, HRQoL domains, and motivational regulation. Based on the current literature, we hypothesise that higher intrinsic motivation and better HRQoL scores are positively associated with greater enjoyment of exercise and PA, beyond the influence of clinical characteristics.

## 2. Materials and Methods

This cross-sectional study was conducted at the Adult Cystic Fibrosis Centre of the Ruhrlandklinik, Essen, Germany. Participants were pwCF aged ≥18 years, with a confirmed diagnosis of CF, based on two defining mutations in the CFTR gene. Recruitment occurred during routine outpatient clinic visits.

After providing their written informed consent, the participants completed standardised and validated questionnaires assessing their (1) motivation for PA and exercise, (2) enjoyment of exercise and PA participation, and (3) HRQoL.

Clinical data were obtained from medical records, including ppFEV_1_, age (years), height (cm), weight (kg), body mass index (BMI, kg/m^2^), pancreatic status (sufficiency/insufficiency), CFTR modulator therapy, presence of cystic fibrosis-related diabetes (CFRD), history of transplantation (lung [LuTx] or liver [LTx]), and *CFTR* genotype.

Ethical approval was obtained from the Medical Ethics Committee of the University Duisburg-Essen (24-11951-BO).

### 2.1. Questionnaires

#### 2.1.1. Enjoyment

PA and exercise enjoyment were assessed using the German version of the Physical Activity Enjoyment Scale (PACES) [23]. Based on Motl et al.’s adaptation, the PACES questionnaire includes 16 items, each rated on a 5-point Likert scale (from 1 = strongly disagree to 5 = strongly agree) [24]. Example items include “I find physical activity pleasurable” and “Physical activity gives me energy”. The German version has shown excellent internal consistency (Cronbach’s α = 0.92–0.93) [23].

Participants were asked to reflect on their typical experiences with PA and/or exercise over the past month. The total scores were calculated by adding the responses to each item (range: 16 to 80), with higher scores reflecting greater enjoyment.

#### 2.1.2. Motivation

Motivation for PA and exercise was assessed using the German version of the Behavioural Regulation in Exercise Questionnaire-2 (BREQ-2) which demonstrates acceptable internal consistency across its subscales (Cronbach’s α = 0.62–0.90) [25]. Based on SDT, the BREQ-2 consists of 19 items rated on a 5-point Likert scale (from 1 = not true for me to 5 = very true for me) [26,27]. Five different regulation types are covered: amotivation (4 items; e.g., “I don’t see why I should have to exercise”), external regulation (4 items; e.g., “I exercise because other people say I should”), introjected regulation (3 items; e.g., “I feel guilty when I don’t exercise”), identified regulation (4 items; e.g., “I value the benefits of exercise”), and intrinsic motivation (4 items; e.g., “I find exercise a pleasurable activity”) [27].

Participants were instructed to respond according to their usual PA and exercise habits. Each subscale score was calculated by averaging the corresponding item responses, with higher scores indicating stronger endorsement of that type of regulation.

The Relative Autonomy Index (RAI) was calculated using the following formula: RAI = (3 × intrinsic motivation) + (2 × identified regulation) + (−1 × introjected regulation) + (−2 × external regulation) + (−3 × amotivation). Higher RAI scores indicate a greater degree of autonomous motivation.

The BREQ-2 and PACES questionnaires both use the term exercise. In this study, the instruments were applied in their original form, but the term exercise was interpreted broadly. Depending on individual health status and opportunities, participants may have referred either to structured exercise (e.g., aerobic or resistance training in rehabilitation or leisure settings) or to PA in daily life. To account for this variability, the terms PA and exercise are used throughout this manuscript.

#### 2.1.3. Health-Related Quality of Life

HRQoL was assessed using the revised German version of the Cystic Fibrosis Questionnaire (CFQ-R) [28]. The CFQ-R is a disease-specific questionnaire that has been validated for use in both research and clinical settings.

The questionnaire includes 50 items, grouped into 12 domains covering physical functioning, vitality, emotional functioning, social functioning, role limitations, nutrition, treatment burden, health perception, body image, weight, respiratory symptoms, and digestive symptoms. Each domain was analysed independently, and scores were standardised on a scale from 0 to 100, with higher values indicating better quality of life in that domain. The CFQ-R provides information on the impact of CF on multiple aspects of daily living [29]. In this study, only the adult version was used to assess the quality of life among the adult pwCF. All 12 CFQ-R domains were included to examine their association with PA and exercise enjoyment.

### 2.2. Statistics

All data are presented as mean ± standard deviation (SD) and 95% confidence intervals (CI) values. Descriptive statistics were calculated, and the data distribution was assessed using the Shapiro–Wilk test and Q–Q plots. Spearman’s rank correlation coefficients were used to examine the relationships between clinical outcomes, motivation, and HRQoL domains.

To identify predictors of enjoyment, a three-stage stepwise linear regression analysis was performed. In the first stage, the clinical parameters were included as predictors. In the second stage, the model was expanded to include the HRQoL domains. In the final stage, motivational subscales from the BREQ-2 were included alongside the clinical parameters and HRQoL domains.

Among the lung function parameters, ppFEV_1_ was selected for the regression analysis as it is widely considered to be a more sensitive marker of disease progression and the main prognostic factor in CF, particularly in the context of CFTR modulator therapy. However, given the strong correlation between ppFEV_1_ and ppFVC, including both variables in the models may introduce multicollinearity.

Statistical significance was set at *p* < 0.05 (two-tailed). All statistical analyses were performed using SPSS version 29.0 (IBM Corp., Armonk, NY, USA).

## 3. Results

**Figure 1:**
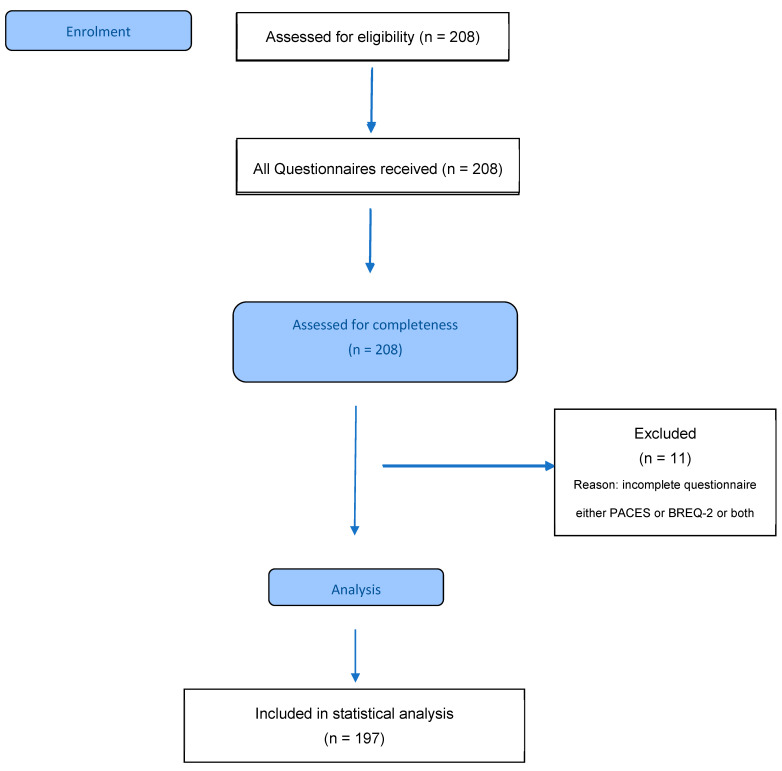
Participant flow diagram. PACES: Physical Activity Enjoyment Scale; BREQ-2: Behavioural Regulation in Exercise Questionnaire-2.

A total of 208 pwCF were willing to participate in this study. Due to incomplete questionnaire data (either the CFQ-R or BREQ-2 or both), only 197 participants were included in the statistical analysis (Figure 1).

### 3.1. Demographic and Clinical Data

The demographic and clinical data of the participants are presented in Table 1. The final sample (n = 197) had a mean age of 36.6 years (SD 11.9), with a slight male dominance (59.4%). The mean BMI was 24.0 kg/m^2^ (SD 4.5), and the ppFEV_1_ was 70.6 (SD 22.4). Most participants (80.7%) received CFTR modulator therapy, and 86.9% had pancreatic insufficiency. Genetic analysis revealed that 47.7% of participants were homozygous and 41.1% were heterozygous for the F508del mutation.

### 3.2. CFQ-R Domain Scores

The results of the HRQoL assessment for all 12 CFQ-R domains are presented in Table 2. The highest mean scores were observed for eating disturbances (mean 91.7, SD 16.0) and digestive symptoms (mean 83.7, SD 29.2). High mean scores were also found for emotional functioning (mean 80.3, SD 19.8), role functioning (mean 79.6, SD 21.4), weight (mean 78.8, SD 20.7), respiratory symptoms (mean 78.5, SD 18.6), treatment burden (mean 78.5, SD 19.4), physical functioning (mean 77.1, SD 22.5), body image (mean 75.1, SD 21.1), and social functioning (mean 74.3, SD 20.5).

The lowest mean scores were observed for health perceptions (mean 65.3, SD 22.6) and vitality (mean 58.0, SD 21.8).

### 3.3. Motivation and Enjoyment

The participants’ motivational profiles and enjoyment of PA are described in Table 3. The highest mean scores among the BREQ-2 subscales were observed for intrinsic regulation (mean 3.6, SD 1.2) and identified regulation (mean 3.5, SD 1.0). Lower scores were observed for introjected regulation (mean 2.1, SD 0.9), external regulation (mean 1.4, SD 0.7) and amotivation (mean 1.3, SD 0.6).

The Relative Autonomy Index (RAI), which measured overall self-determination to engage in PA, showed a mean score of 9.0 (SD 6.9).

For PA and exercise enjoyment, as measured by the PACES total score, the mean score was 65.5 (SD 11.2).

### 3.4. Regression Analysis Results

#### 3.4.1. Model 1: Clinical Parameters

The first regression model included the following clinical covariates: age, sex, BMI, ppFEV_1_, and the use of CFTR modulators (Table 4a). However, this model only explained a small proportion of the variance in enjoyment (R^2^ = 0.023, adjusted R^2^ = 0.020, F(5,191) = 0.90, *p* = 0.370). Of these predictors, only sex and ppFEV_1_ were significantly associated with enjoyment. Specifically, lower enjoyment scores were associated with being female (β = −3.864, 95% CI −7.38 to −0.35, *p* = 0.032), while higher ppFEV_1_ was positively associated with higher enjoyment scores (β = 0.124, 95% CI 0.04 to 0.21, *p* = 0.003).

#### 3.4.2. Model 2: Clinical Parameters and CFQ-R Domains

The second model added CFQ-R domain scores in conjunction with clinical variables (Table 4b). This model accounted for a significantly higher proportion of the variance (R^2^ = 0.310, adjusted R^2^ = 0.246, F(14,182) = 5.86, *p* < 0.001). The significant predictors in this model were physical functioning (β = 0.181, 95% CI 0.09 to 0.27, *p* < 0.001), vitality (β = 0.114, 95% CI 0.01 to 0.23, *p* = 0.039), and respiratory symptoms (β = −0.108, 95% CI −0.21 to −0.003, *p* = 0.045). Higher physical functioning and vitality scores were associated with greater enjoyment, whereas more severe respiratory symptoms were associated with lower enjoyment.

#### 3.4.3. Model 3: Clinical Parameters, CFQ-R Domains, and BREQ-2 Motivation Variables

The third model also incorporated motivational variables from the BREQ-2 (Table 4c). This comprehensive model explained the largest proportion of the variance in enjoyment (R^2^ = 0.684, adjusted R^2^ = 0.645, F(19,177) = 20.08, *p* < 0.001). The key predictors were health perception (β = 0.081, 95% CI 0.004 to 0.16, *p* = 0.038), external regulation (β = −1.932, 95% CI −3.67 to −0.19, *p* = 0.030), and intrinsic regulation (β = 6.228, 95% CI 4.52 to 7.94, *p* < 0.001). Higher intrinsic motivation was strongly associated with greater enjoyment, whereas external regulation was negatively associated with it.

## 4. Discussion

This study aimed to explore the predictors of enjoying PA and exercise among adult pwCF, focusing on HRQoL, motivational regulation, and clinical factors. Our hierarchical regression analysis of data from 197 adult pwCF revealed that motivational factors, particularly intrinsic motivation, were substantially more predictive of exercise enjoyment than clinical parameters or most HRQoL domains. These findings provide valuable information on the psychological factors that influence PA enjoyment in CF, highlighting motivation as a potentially modifiable target. Enjoyment is a key affective determinant of sustained engagement in exercise, particularly in the context of CF, for which the treatment burden is high and adherence to regular PA and exercise is often suboptimal [1].

In line with SDT, intrinsic motivation was found to be the strongest positive predictor of enjoyment, demonstrating a large effect size in the present study [8,11,12]. This finding is supported by a substantial body of research indicating that behaviours driven by intrinsic motivation, or performed out of inherent interest or enjoyment, are more likely to result in positive emotional experiences and sustained participation [7,8]. However, it should be noted that our cross-sectional design does not allow us to draw definitive conclusions about whether enjoyable experiences foster intrinsic motivation or whether intrinsic motivation leads to greater enjoyment.

In pwCF, intrinsic motivation can indicate a transition from compliance-based to autonomy-based engagement in PA and exercise, which is particularly important given the long-term nature of CF management. These results are consistent with previous studies in healthy populations and clinical contexts, which emphasise that intrinsic enjoyment of PA is a key predictor of long-term adherence [7,13,14].

In contrast, external regulation was significantly and negatively associated with enjoyment, but to a lesser extent. This finding supports the idea that motivation driven by external demands, such as pressure from clinicians or family members, can undermine the emotional experience of PA. While this is consistent with SDT predictions, the moderate effect size suggests that, while detrimental, the influence of external regulation may be less significant than the positive effects of intrinsic motivation.

Of the HRQoL domains, only health perception was found to be significantly and positively associated with enjoyment in the final model. This suggests that individuals who perceive their health status more favourably also experience greater enjoyment of PA and exercise [2,3,30]. However, the small effect size indicates that although the relationship is statistically significant, it may have limited practical significance. One possible explanation is that perceived health enhances confidence and perceived competence, both of which are essential to promoting intrinsic motivation, as proposed by SDT [8,12].

Notably, other HRQoL domains, such as respiratory symptoms, treatment burden, emotional functioning, and vitality, did not emerge as significant predictors in the final model. These domains showed significance in Model 2 but became non-significant when motivational variables were added in Model 3, therefore this finding requires cautious interpretation. This pattern suggests potential mediation effects rather than a true lack of association between these variables. This apparent lack of association is somewhat unexpected, particularly given the well-documented role of respiratory symptom and treatment burden in hindering PA in pwCF [2].

In an era of highly effective CFTR modulators, respiratory symptom scores on the CFQ-R have improved significantly, reflecting reductions in baseline dyspnoea and sputum burden [31,32]. Nevertheless, our results suggest that better respiratory function alone does not directly lead to a greater PA and exercise enjoyment or higher capacity for cardiorespiratory exercise Although 80.7% of our participants received CFTR modulator therapy, modulator use itself was not a significant predictor of enjoyment. This may be due to the high prevalence in our sample, which limited variability. We did not directly assess the impact of modulators on enjoyment as this was not the primary aim of our study. Our cross-sectional design also means that we cannot establish temporal relationships between modulator use and outcomes.

Notably, genetic factors including F508del mutation status did not emerge as significant predictors of exercise enjoyment in the regression models. This suggests that motivational and quality of life factors may be more influential determinants of PA enjoyment than specific CFTR genotypes. While different CFTR mutations are associated with varying disease severity and clinical phenotypes, our findings indicate that the psychological experience of exercise enjoyment may be relatively independent of underlying genetic variations. However, this should be interpreted cautiously given that our sample was predominantly composed of pwCF with F508del mutations, which may have limited our ability to detect genotype-related differences.

Other motivational subtypes, such as identified and introjected regulation, showed no significant association with enjoyment, and negligible effect sizes. While these regulation types represent partially internalised motives, they may not produce strong emotional outcomes in this population unless they are fully integrated or experienced as intrinsically rewarding. However, this result should be interpreted with caution, as moderate correlations between the BREQ-2 subscales may have introduced multicollinearity, which could have obscured the unique contribution of partially internalised regulation types such as identified motivation [27].

The lack of significance for amotivation, introjected regulation and identified regulation may be due to multicollinearity between BREQ-2 subscales, or to the dominant effect of intrinsic motivation within the model. Further longitudinal research is required to examine how these forms of regulation interact over time and whether shifts towards more autonomous motivation result in increased enjoyment and commitment.

From a clinical perspective, these findings highlight the importance of targeting motivational quality rather than relying solely on extrinsic incentives or structured compliance to improve adherence. However, translating these findings into clinical practice presents challenges.

Interventions designed to promote PA in pwCF should focus on creating autonomy-supportive environments that encourage personal choice, recognise individual progress, and foster a sense of relatedness and belonging [5,16]. Specific strategies might include collaborative goal setting, offering exercise options that align with patient preferences, and incorporating enjoyable activities into treatment routines. However, implementing such approaches requires significant changes to the clinical culture and may encounter obstacles such as time constraints, reimbursement issues and the need for provider training.

Furthermore, the communication style adopted and the broader clinical environment play a crucial role in shaping the motivation of people with CF. Autonomy-supportive communication, which is characterised by empathy, shared decision-making and providing meaningful rationales for therapeutic recommendations, has been shown to promote self-determined motivation [5,11,16,33]. Although these strategies are well established in theory, there is limited empirical evidence for their effectiveness in CF populations.

In the context of CF care, the entire multidisciplinary team plays a role in fostering intrinsic motivation and volitional aspects. Techniques such as developing personalised action plans, identifying values associated with physical activity (PA) and exercise, and anticipating barriers (e.g., through implementation intentions or coping plans) can support volitional processes and bridge the gap between intention and behaviour [34]. Care teams can improve perceived autonomy and competence by addressing potential barriers together and providing structure without pressure on individuals [30]. This approach can help create an environment that fosters enjoyment and long-term commitment. Improving pwCF’s perception of their health progress, even incrementally, may also encourage more positive emotional responses to PA.

There are several important limitations of this study that must be acknowledged. First, the cross-sectional design restricts the ability to make causal inferences. It is not possible to definitively determine whether higher intrinsic motivation leads to greater enjoyment or whether those who naturally enjoy PA subsequently develop more intrinsic motivation. It is likely that there are bidirectional relationships, and longitudinal designs with multiple time points are needed to establish causal pathways and identify optimal intervention timing.

Second, potential selection bias may have affected generalisability of the results. Participants were recruited from a single clinical centre and were predominantly adults with moderate disease severity, relatively high baseline HRQoL scores, and predominantly intrinsic motivation profiles. Individuals willing to participate in PA research may already be more motivated or engaged, which limits the applicability of the findings to less motivated individuals who might benefit most from interventions.

Most of the participants received CFTR modulator therapy. These factors suggest that the findings may not be applicable to individuals with more advanced disease, paediatric patients, or those not yet receiving modulator treatment. Including a more diverse range of clinical phenotypes in future research could improve the external validity of these findings.

An a priori power analysis was not conducted to determine sample size. However, our final sample size of 197 with 19 predictors (approximately 10.4 observations per predictor) satisfies the common ‘≥10 per predictor’ heuristic and exceeds Green’s guideline for testing individual predictors (N ≥ 104 + m = 123). However, for testing the overall model, Green recommends N ≥ 50 + 8m = 202, and our sample size is five participants short of this threshold [35]. Consequently, the results should be interpreted with appropriate caution, although the shortfall is minimal. A larger sample size of more than 202 participants would nonetheless have provided greater statistical power and further reduced the risk of overfitting.

Additionally, as all data were self-reported, including the motivation and enjoyment measures, there is a possibility of response bias (e.g., social desirability or common method bias). Future studies should incorporate objective activity trackers or clinical outcome data to complement self-reported enjoyment, examining how motivation relates to actual exercise behaviour or health outcomes over time.

Motivation and enjoyment were assessed using self-reporting. Therefore, the findings reflect the participants’ perceptions of their PA and exercise behaviour and may be influenced by recall or reporting bias. Additionally, differences in how participants understood the term ‘exercise’ cannot be ruled out. This study did not assess unmeasured confounders, such as depression, anxiety, social support, and environmental factors. These factors may influence both motivation and enjoyment and should be explored in further research.

## 5. Conclusions

Among adult pwCF, intrinsic motivation was found to be the strongest predictor of PA enjoyment, while external regulation showed negative associations and health perceptions contributed modestly to the model. These findings provide initial empirical support for applying SDT principles to CF care through autonomy-supportive, personalised, and competence-enhancing strategies. However, the cross-sectional nature of our data means that causal relationships should be interpreted with caution. As a potentially modifiable affective outcome, enjoyment should be considered a target in behavioural interventions aimed at improving long-term adherence to PA, although longitudinal evidence of modifiability is needed. Future research priorities should include longitudinal validation of these relationships, the development and testing of SDT-based interventions in CF populations, and the investigation of barriers to implementation in clinical settings.

## Figures and Tables

**Table 1:** Demographic data and clinical outcomes.

	pwCF (n = 197)	
**Charcteristics**		**95.0% CI (LL/UL)**
**Demographics**		
male, n (%)	117 (59.4)	
age (years), mean (SD)	36.6 (11.9)	34.8/38.4
height (cm), mean (SD)	172.7 (9.2)	171.3/174.1
weight (kg), mean (SD)	70.8 (15.1)	69.5/74.1
BMI (kg/m^2^), mean (SD)	24.0 (4.5)	23.5/24.8
**Pulmonary Function**		
ppFEV_1_ (% predicted), mean (SD)	70.6 (22.4)	67.3/74.0
ppFVC (% predicted), mean (SD)	87.5 (18.6)	84.7/90.3
**Genetics**		
del F508 homozygous, n (%)	94 (47.7)	
del F508 heterozygous, n (%)	81 (41.1)	
other mutations, n (%)	21 (10.7)	
**Treatment**		
CFTR-Modulator (mono, dual or triple combination), n (%)	159 (80.7)	
**Comorbidities**		
Pancreatic insufficiency, n (%)	171 (86.9)	
CFRD, n (%)	17 (8.6)	
Pseudomonas aeruginosa, n (%)	102 (51.8)	
**Transplantation**		
LuTx, n (%)	1 (0.5)	
LTx, n (%)	8 (4.2)	

Data are presented as mean (SD) and 95% CI for continuous variables, and as n (%) for categorical variables. 95% CI = confidence interval (lower to upper limit); CFRD = Cystic Fibrosis-Related Diabetes, LuTx = lung transplantation, LTx = liver transplantation; BMI = Body Mass Index; ppFEV_1_ = percent predicted forced expiratory volume in 1 s; ppFVC = percent predicted forced vital capacity.

**Table 2:** Cystic Fibrosis Questionnaire-Revised (CFQ-R) domain scores in adults pwCF.

	pwCF (n = 197)	
	**Mean ± SD**	**95.0% CI (LL/UL)**
**Quality of life domains**		
Physical Functioning	77.1 (22.5)	73.7/80.5
Vitality	58.0 (21.8)	54.7/61.3
Emotional State	80.3 (19.8)	77.3/83.3
Eating Disturbances	91.7 (16.0)	89.2/94.1
Treatment Burden	78.5 (19.4)	75.5/81.5
Social Functioning	74.3 (20.5)	71.2/77.5
Body Image	75.1 (21.1)	72.5/78.9
Role Functioning	79.6 (21.4)	76.3/82.8
**Health Perception scale**		
Health	65.3 (22.6)	61.8/68.7
**Symptoms Domains**		
Digestive Symptoms	83.7 (29.2)	79.2/88.1
Respiratory Symptoms	78.5 (18.6)	75.7/81.3
Weight	78.8 (20.7)	75.6/81.9

Data are presented as mean (SD). 95% CI = confidence interval (lower to upper limit); CFQ-R = Cystic Fibrosis Questionnaire-Revised; CFQ-R scoring: Domain scores range from 0–100, with higher scores indicating better health-related quality of life.

**Table 3:** BREQ-2 and PACES questionnaire results in pwCF.

	pwCF (n = 197)	
	**Mean (SD)**	**95.0% CI (LL/UL)**
**BREQ-2 Subscales**		
Mean Amotivation	1.3 (0.6)	1.2/1.4
Mean External Regulation	1.4 (0.7)	1.3/1.5
Mean Introjected Regulation	2.1 (0.9)	2.0/2.3
Mean Identified Regulation	3.5 (1.0)	3.3/3.6
Mean Intrinsic Regulation	3.6 (1.2)	3.5/3.8
Relative Autonomy Index (RAI)	9.0 (6.9)	8.0/10.1
**PACES**		
Enjoyment (total score)	65.5 (11.2)	63.8/67.2

Data are presented as mean (SD); 95% CI = confidence interval (lower to upper limit); BREQ-2 = Behavioral Regulation in Exercise Questionnaire-2; PACES = Physical Activity Enjoyment Scale; BREQ-2 scoring: each subscale score calculated as mean of respective items (range 1–5), with higher scores indicating stronger regulation type. The RAI represents the degree of self-determined motivation, with higher positive values indicating more autonomous motivation and negative values indicating more controlled motivation; PACES scoring: Sum of all 18 items, with higher scores indicating greater physical enjoyment.

**Table 4:** (a) Hierarchical linear regression predicting enjoyment—Model 1. (b) Hierarchical linear regression predicting enjoyment—Model 2. (c) Hierarchical linear regression predicting enjoyment—Model 3.

**(a)**							
**Dependent Variable: Enjoyment**							
**Predictor**						**95% CI**	
**Coefficients**	**b**	**SE**	**β**	**T**	***p***	**LL**	**UL**
(constant)	60.526	6.208	---	9.750	<0.001	48.266	72.786
Age	0.074	0.079	0.079	0.94	0.349	−0.082	0.231
Sex	−3.864	1.791	−0.169	−2.158	**0.032**	−7.401	−0.328
BMI	−0.21	0.198	−0.086	−1.065	0.288	−0.601	0.18
ppFEV_1_	0.124	0.04	0.249	3.058	**0.003**	0.044	0.204
CFRT Modulator	0.033	1.274	0.002	0.026	0.979	−2.484	2.550
**(b)**							
**Dependent Variable: Enjoyment**							
**Predictor**						**95% CI**	
**Coefficients**	**b**	**SE**	**β**	**T**	***p***	**LL**	**UL**
(constant)	45.568	8.611	---	5.292	<0.001	28.551	62.586
Age	0.089	0.074	0.094	1.201	0.232	−0.057	0.234
Sex	−1.288	1.672	−0.056	−0.77	0.443	−4.593	2.017
BMI	0.068	0.188	0.028	0.359	0.72	−0.304	0.44
ppFEV_1_	0.066	0.041	0.132	1.602	0.111	−0.015	0.147
CFRT Modulator	0.062	1.153	0.004	0.054	0.957	−2.216	2.340
Physical Functioning	0.181	0.054	0.364	3.381	**<0** **.001**	0.075	0.287
Vitality	0.114	0.055	0.219	2.080	**0.039**	0.006	0.222
Emotion	0.034	0.058	0.06	0.58	0.563	−0.082	0.15
Eating disturbances	−0.023	0.059	−0.033	−0.384	0.701	−0.139	0.094
Treatment Burden	−0.065	0.049	−0.114	−1.337	0.183	−0.162	0.031
Health Perceptions	0.056	0.057	0.113	0.989	0.324	−0.056	0.168
Social Functioning	0.051	0.056	0.094	0.925	0.356	−0.058	0.161
Body Image	0.038	0.045	0.072	0.858	0.392	−0.05	0.127
Role Functioning	−0.046	0.055	−0.089	−0.839	0.403	−0.154	0.062
Digestive Symptoms	−0.03	0.03	−0.078	−0.976	0.331	−0.09	0.03
Respiratory Symptoms	−0.108	0.053	−0.18	−2.025	**0.045**	−0.213	−0.003
Weight	−0.012	0.041	−0.023	−0.304	0.761	−0.092	0.068
**(c)**							
**Dependent Variable: Enjoyment**							
**Predictor**						**95% CI**	
**Coefficients**	**b**	**SE**	**β**	**T**	***p***	**LL**	**UL**
(constant)	42.259	7.209	---	5.862	<0.001	28.008	56.509
Age	0.039	0.05	0.041	0.777	0.439	−0.06	0.137
Sex	0.356	1.142	0.016	0.312	0.755	−1.9	2.613
BMI	−0.007	0.129	−0.003	−0.058	0.954	−0.261	0.247
ppFEV_1_	−0.004	0.028	−0.007	−0.125	0.901	−0.059	0.052
CFRT Modulator	0.155	0.782	0.009	0.199	0.843	−1.39	1.701
Physical Functioning	0.045	0.038	0.091	1.181	0.239	−0.03	0.12
Vitality	−0.004	0.038	−0.007	−0.097	0.923	−0.079	0.071
Emotion	−0.022	0.039	−0.039	−0.551	0.582	−0.1	0.056
Eating disturbances	0.004	0.04	0.006	0.108	0.914	−0.074	0.083
Treatment Burden	−0.03	0.033	−0.053	−0.918	0.36	−0.096	0.035
Health Perceptions	0.081	0.038	0.162	2.097	**0.038**	0.005	0.157
Social Functioning	−0.007	0.038	−0.013	−0.189	0.85	−0.083	0.069
Body Image	0.022	0.031	0.042	0.716	0.475	−0.039	0.083
Role Functioning	0.005	0.037	0.01	0.133	0.894	−0.069	0.079
Digestive Symptoms	−0.021	0.021	−0.056	−1.033	0.304	−0.062	0.019
Respiratory Symptoms	0.008	0.038	0.014	0.221	0.825	−0.067	0.084
Weight	−0.013	0.027	−0.023	−0.461	0.646	−0.067	0.042
Mean Amotivation	−1.875	1.143	−0.1	−1.641	0.103	−4.134	0.383
Mean External Regulation	−1.932	0.88	−0.114	−2.195	**0.03**	−3.671	−0.192
Mean Introjected Regulation	0.178	0.741	0.014	0.24	0.811	−1.287	1.643
Mean Identified Regulation	−0.066	0.945	−0.006	−0.07	0.944	−1.934	1.802
Mean Intrinsic Regulation	6.228	0.878	0.639	7.091	**<0** **.001**	4.492	7.964

Remark: model 1: N = 164; R2 = 0.091; corr. R2 = 0.062; F (5,159); *p* < 0.009. Remark: model 2: N = 164; R2 = 0.349; corr. R2 = 0.274; F (17,147); *p* < 0.001. Weight refers to the CFQ-R Weight domain score (0–100 scale), not body weight in kilograms. Remark: model 3: N = 164; R2 = 0.718; corr. R2 = 0.674; F (22,142); *p* < 0.001. Weight refers to the CFQ-R Weight domain score (0–100 scale), not body weight in kilograms. Significant results are shown in bold.

## Data Availability

Due to data protection laws, the data presented in this study are only available upon request and in justified cases, from the corresponding authors.
